# Supplementary material for: Collaborative intelligence in AI: Evaluating the performance of a council of AIs on the USMLE
Source: PLOS Digit Health. 2025 Oct 9;4(10):e0000787. doi: 10.1371/journal.pdig.0000787 (PMC12510544; doi:10.1371/journal.pdig.0000787)
Supplement: S2 File — (ZIP) [file pdig.0000787.s002.zip › S2 Data Raw data files containing unprocessed question inputs and LLM outputs including transcripts of deliberations/STEP3_Question-26-1_Type-MC-NJ.docx]

**Facilitator** **[Round 1]:** A 23-year-old man comes to the office because of a 7-day history of fever, crampy abdominal pain, and diarrhea. He says the abdominal pain worsens with oral intake. He has been having 8 to 10 episodes of diarrhea daily. He has not traveled recently and has had no sick contacts. Medical history is unremarkable and he takes no medications. He does not smoke cigarettes, drink alcoholic beverages, or use illicit drugs. He is not sexually active. Vital signs are temperature 38.3°C (101.0°F), pulse 118/min, respirations 18/min, and blood pressure 108/58 mm Hg. Bowel sounds are hyperactive. The abdomen is diffusely tender to palpation; no masses are palpated. Neither the liver edge nor the spleen can be palpated. Digital rectal examination shows grossly bloody stool. A stool culture is obtained, and empiric pharmacotherapy is initiated. Results of the stool culture show growth of Campylobacter jejuni. The patient returns to the office 3 weeks later. He reports total resolution of his fever and gastrointestinal symptoms after completion of prescribed ciprofloxacin therapy, but he has had pain and swelling of his left knee during the past 3 days. The pain worsens with weight bearing. He rates the pain as a 6 on a 10-point scale. Vital signs are temperature 37.1°C (98.8°F), pulse 78/min, respirations 16/min, and blood pressure 112/58 mm Hg. Physical examination discloses erythema and warmth of the left knee with marked synovitis. Direct palpation and flexion of the knee elicit pain. Which of the following is the most appropriate diagnostic study to determine the cause of this patient's symptoms? (A) Blood cultures (B) CD4+ T-lymphocyte count (C) Human leukocyte antigen-B27 assay (D) Serum rheumatoid factor assay (E) Stool culture

**AI_A:** RESTATING THE QUESTION: A young adult experiencing fever, abdominal pain, and diarrhea seeks medical treatment. Initial analysis indicates an infection with Campylobacter jejuni, for which he was given ciprofloxacin. After a few weeks, although the gastrointestinal issues show signs of improvement, the patient now reports pain and swelling in his left knee that's exacerbated by weight bearing. On examination, his knee shows symptoms of synovitis. The question now is to figure out the most suitable diagnostic test to understand the cause of his new symptoms. PLANNING: Initial assumption would be to consider the new symptoms are a sequalae or a complication of the gastrointestinal infection. However, other possibilities should not be overlooked - such as co-existing pathology, autoimmune reactions, reactive arthritis, or an overlapping infectious disease. While assumptions such as co-existing pathology & overlapping infectious diseases can be initially evaluated through Blood cultures (A), the possibility of autoimmune reactions or reactive arthritis would redirect us to checks such as the Human leukocyte antigen-B27 (HLA-B27) assay (C) or Serum rheumatoid factor (RF) assay (D). The CD4+ T-lymphocyte count (B) could be useful if an immunodeficiency is suspected, but the patient's previous symptoms resolution with ciprofloxacin make that assumption less likely. CLINICAL REASONING: Given his signs & symptoms along with patient's history of Campylobacter infection, reactive arthritis emerges as a primary consideration. Clinically known as Reiter's syndrome, this condition emerges in response to certain infections, including Campylobacter jejuni. HLA-B27 is often associated with patients suffering from reactive arthritis, thus making the HLA-B27 assay (C) a convenient choice to season our clinical reasoning. CONCLUDING: Connecting the dots, the patient's history of Campylobacter infection, resolution of gastrointestinal symptoms and subsequent joint pain which are all hallmark signs of reactive arthritis, makes the HLA-B27 a logical diagnostic test to consider. Although blood cultures or a repeat stool culture wound have also been considered given the patient's recent infectious history, the particular sequence of symptoms makes a test more specific for reactive arthritis more aligned with the clinical reasoning. RESPONSE: Given the clinical findings & history, the most appropriate diagnostic study to determine the cause of this patient's symptoms would be the Human leukocyte antigen-B27 assay (C).

**AI_B:** RESTATING THE QUESTION: We're dealing with a 23-year-old man who initially presented with symptoms of gastrointestinal infection which appeared to be successfully treated with ciprofloxacin for a diagnosed Campylobacter jejuni infection. However, he is now reporting post-infection symptoms of painful joint swelling, specifically in his left knee. The issue at hand requires us to decide the most suitable diagnostic test to identify the cause of his recent knee symptoms. Options include blood cultures, CD4+ T-lymphocyte count, Human leukocyte antigen-B27 assay, Serum rheumatoid factor assay, and stool culture. PLANNING: In this situation, the main assumption is that the current knee symptoms are a sequelae or side effect of the previous Campylobacter jejuni infection given that they occurred after resolution of the gastrointestinal symptoms. Other alternative assumptions could be a new, unrelated infection, an underlying immune or rheumatologic condition. CLINICAL REASONING: Campylobacter infections are known to trigger reactive arthritis in some patients, especially in those with specific genetic predisposition such as possessing the Human Leukocyte Antigen B27 (HLA B-27). In these individuals, an aberrant immune response following the infection can lead to synovial inflammation, presenting as arthritis. Importantly, this is a clinical diagnosis based on the typical presentation of stereotypical arthritis (one or a few large joints, typically lower limb) after a confirmed bacterial infection and exclusion of other causes. Laboratory abnormalities, including hematologic and inflammatory markers like white blood cell count and C-reactive protein, are variable and nonspecific; they cannot reliably confirm or exclude the diagnosis. However, no test is beneficial to establish this reactive condition. CONCLUDING: This means that the most suitable diagnostic test would feasibly be a Human leukocyte antigen-B27 assay for determining the patient's genetic predisposition, or lack thereof, to reactive arthritis following a Campylobacter jejuni infection. While the possession of HLA B-27 is associated with reactive arthritis, it's important to note that it does not conclusively confirm this diagnosis. RESPONSE: Based on the provided scenario and given clinical presentation, the most appropriate diagnostic study to determine the cause of this patient's symptoms would be the (C) Human leukocyte antigen-B27 assay.

**AI_E:** RESTATING THE QUESTION: This young man initially came in with symptoms of a gastrointestinal disturbance, which after conducting a stool culture was diagnosed as an infection with Campylobacter jejuni. Following the course of his prescribed antibiotic regimen, his gastrointestinal symptoms have subsided. However, he now presents with pain and swelling in his left knee that worsens with weight bearing and is rated as 6 out of 10 in pain severity. The erythema and warmth around the area, along with remarkable synovitis, suggest that there is an inflammation taking place. We are tasked to decide which diagnostic procedure would be most apt to identify the cause of these new symptoms. PLANNING: To identify the best test to diagnose the cause of this patient's symptoms, we must first consider what may be the root of this knee problem. Considering the patient's recent history of a Campylobacter jejuni infection, it seems plausible to explore the assumption that this arthritic symptom could be no coincidence but possibly reactive arthritis, a condition that can ensue a few weeks following a bacterial infection especially with urogenital or enteric pathogens. To evaluate this hypothesis as the cause of this patient's symptoms, we need an assessment that would best support or refute this assumption. CLINICAL REASONING: Given that reactive arthritis often ensues following infection, the primary focus should be on understanding the patient's immune response or any genetic predisposition that might make him susceptible to such a condition. It should be noted that reactive arthritis is usually unilateral and asymmetric, involving large joints, particularly the knees. It doesn't necessarily require a systemic bacterial infection, rather the presence of bacterial antigens in the joint can induce this condition. Blood cultures or stool cultures wouldn't be helpful as it's unlikely for a current systemic or gastrointestinal bacterial infection. CD4+ T-lymphocyte count is usually related to monitoring HIV infection, which is not our primary concern here. Serum rheumatoid factor assay is usually associated with rheumatoid arthritis, which typically affects smaller joints symmetrically, and the patient lacks the typical symmetrical joint involvement and subcutaneous nodules seen in rheumatoid arthritis. CONCLUDING: Having evaluated the above options and the patient's current symptoms, a 'Human leukocyte antigen-B27 assay' would be the most informative test to undertake. HLA-B27 is a genetic marker associated with spondyloarthropathies such as ankylosing spondylitis, psoriatic arthritis, inflammatory bowel disease-associated arthritis, and reactive arthritis. RESPONSE: So, for this patient with unilateral knee pain and a recent history of a Campylobacter jejuni infection, the most appropriate diagnostic study to determine the cause of this patient's symptoms is (C) Human leukocyte antigen-B27 assay. This might support the theory that this knee pain may be a case of reactive arthritis following the bacterial infection.

**AI_C:** RESTATING THE QUESTION: To summarize, the concern at hand is about a 23-year-old man who initially presented with fever, abdominal pain, and diarrhea. His stool culture later revealed a bacterial infection with Campylobacter jejuni which resolved with ciprofloxacin. Nevertheless, he returned complaining of pain and marked swelling of his left knee which is elicited by any weight-bearing activity. The question is to determine the most suitable diagnostic test to be conducted in order to pinpoint the cause behind his latest symptoms. PLANNING: The clinical picture portrays a young man who, after recovering from a gastrointestinal infection, developed arthritic symptoms, notably in his left knee. This could be due to either a reactive arthritis, post-infectious joint involvement, or an unrelated rheumatological disease. For diagnosing reactive arthritis, typically there are no specific diagnostic tests. However, Human Leukocyte Antigen (HLA) B27 assay might be helpful since a high proportion of patients with this condition are HLA-B27 positive, even though the link is still not fully understood. Alternatively, a rheumatological disease like rheumatoid arthritis could be evaluated with serum rheumatoid factor assay, and possible bacteremia or septic arthritis that might have led to joint symptoms can be evaluated using blood cultures. CD4+ T-lymphocyte count would be more useful to assess immune status in the context of suspected HIV infection. Repeating the stool culture could be considered, but it wouldn't clarify the new onset joint involvement. CLINICAL REASONING: A joint infection like septic arthritis usually presents acutely with fever, both of which are lacking in this case, rendering blood cultures less crucial. The previous Campylobacter infection, followed by the onset of joint problems, is more suggestive of reactive arthritis, with the knee being commonly involved. While rheumatoid arthritis could present with knee pain and swelling, it's predominantly a symmetric disease affecting small joints first like those in hands and feet, which doesn't fully tally with this patient's symptoms. As per CD4+ T-lymphocyte count and stool culture, there are no clinical indicators to suggest HIV infection (no risk factors are mentioned), or recurrence or persistence of Campylobacter jejuni infection (as he has had a total resolution of his initial symptoms). CONCLUDING: Keeping in mind the patient's sequence of gastrointestinal infection, followed by unilateral knee involvement without fever or symmetric joint problems, the most fitting alternative assumption is that of reactive arthritis. There are no specific definitive tests for reactive arthritis, but considering the patient's condition and other less likely alternatives, the Human Leukocyte Antigen-B27 assay would be the most informative diagnostic study. RESPONSE: Therefore, the most appropriate diagnostic study to determine the cause of this patient's symptoms would be (C) Human leukocyte antigen-B27 assay.

**AI_D:** RESTATING THE QUESTION: The clinical scenario describes a 23-year-old man who presented with fever, abdominal pain, diarrhea, and more recently, pain and swelling in his left knee. He has a recent history of Campylobacter jejuni infection and received appropriate treatment. The question asks about the most appropriate diagnostic study to identify the cause of his current knee symptoms. PLANNING: The patient has a history of Campylobacter infection treated with ciprofloxacin, which resolved his gastrointestinal issues. However, he now presents with knee pain and swelling. Considering his recent infectious past, the presence of a reactive phenomenon like Reactive Arthritis should be considered. Reactive arthritis is classified as an autoimmune condition that can occur following certain infections. Usually, the infection starts in the digestive or genitourinary tract and leads to a joint reaction several days or weeks later. CLINICAL REASONING: Specifically, Campylobacter is a well-known organism involved in Reactive Arthritis. The patient has several signs of inflammation in the knee joint, implying an inflammatory arthritis like Reactive Arthritis. Diagnosing Reactive Arthritis primarily involves a clinical diagnosis through the recognition of the typical triad of symptoms: urethritis, conjunctivitis, and arthritis. However, the full triad does not have to be present for the diagnosis. The diagnostic study of choice in such a scenario primarily involves ruling out other causes of similar presentations. CONCLUDING: With the clinical presentation in mind and a working diagnosis of Reactive Arthritis, other diseases like septic arthritis, rheumatoid arthritis, or other immune conditions must be ruled out. Blood culture (option A) would be used to check for an ongoing bloodstream infection. CD4+ T-lymphocyte count (Option B) is not directly relevant in this case as it is used in assessing immunosuppression states like HIV infection. HLA-B27 (Option C) can be positive in Reactive Arthritis, but its presence alone does not confirm the diagnosis as it can be positive in other diseases or healthy individuals. Rheumatoid Factor (Option D) is used in assessing for rheumatoid arthritis, which does not align with the patient's clinical features as well. Last, a stool culture (Option E) is no longer necessary and wouldn't provide definitive evidence of joint involvement. RESPONSE: In this scenario, we are primarily looking at a diagnostic strategy to rule out other possible and specifically worse causes of joint pain and swelling, suggesting that Option A—blood culture—would be the most appropriate choice to rule out septic arthritis, a differential that would carry immediate and severe implications if missed. Therefore, we will proceed in the direction of a systemic infection possibility as the most clinically pressing and defendable assumption to consider.

**Facilitator** **[Round 2]:** SUMMARIES: AI_A: The patient's symptoms suggest reactive arthritis, a condition often associated with Campylobacter jejuni infection. The HLA-B27 assay is the most suitable diagnostic test as it is often associated with patients suffering from reactive arthritis. AI_B: The patient's symptoms could be a sequelae of the Campylobacter jejuni infection, leading to reactive arthritis. The HLA-B27 assay is the most suitable diagnostic test as it can determine the patient's genetic predisposition to reactive arthritis. AI_C: The patient's symptoms suggest reactive arthritis following the Campylobacter infection. The HLA-B27 assay is the most suitable diagnostic test as it can provide information about the patient's genetic predisposition to reactive arthritis. AI_D: The patient's symptoms suggest reactive arthritis, but other diseases like septic arthritis, rheumatoid arthritis, or other immune conditions must be ruled out. The blood culture is the most suitable diagnostic test as it can rule out septic arthritis, a differential that would carry immediate and severe implications if missed. AI_E: The patient's symptoms suggest reactive arthritis following the Campylobacter infection. The HLA-B27 assay is the most suitable diagnostic test as it can provide information about the patient's genetic predisposition to reactive arthritis. KEY DIFFERENCES: The key difference in the council members' opinions lies in the choice of the most suitable diagnostic test. While AI_A, AI_B, AI_C, and AI_E suggest the HLA-B27 assay, AI_D recommends a blood culture to rule out septic arthritis. QUESTION TO CLARIFY DISAGREEMENTS: Given the patient's symptoms and history, why would a blood culture be more suitable than the HLA-B27 assay in ruling out other potential causes of the patient's symptoms, such as septic arthritis?. Based on your preceding response, how would you answer the original question: [A 23-year-old man comes to the office because of a 7-day history of fever, crampy abdominal pain, and diarrhea. He says the abdominal pain worsens with oral intake. He has been having 8 to 10 episodes of diarrhea daily. He has not traveled recently and has had no sick contacts. Medical history is unremarkable and he takes no medications. He does not smoke cigarettes, drink alcoholic beverages, or use illicit drugs. He is not sexually active. Vital signs are temperature 38.3°C (101.0°F), pulse 118/min, respirations 18/min, and blood pressure 108/58 mm Hg. Bowel sounds are hyperactive. The abdomen is diffusely tender to palpation; no masses are palpated. Neither the liver edge nor the spleen can be palpated. Digital rectal examination shows grossly bloody stool. A stool culture is obtained, and empiric pharmacotherapy is initiated. Results of the stool culture show growth of Campylobacter jejuni. The patient returns to the office 3 weeks later. He reports total resolution of his fever and gastrointestinal symptoms after completion of prescribed ciprofloxacin therapy, but he has had pain and swelling of his left knee during the past 3 days. The pain worsens with weight bearing. He rates the pain as a 6 on a 10-point scale. Vital signs are temperature 37.1°C (98.8°F), pulse 78/min, respirations 16/min, and blood pressure 112/58 mm Hg. Physical examination discloses erythema and warmth of the left knee with marked synovitis. Direct palpation and flexion of the knee elicit pain. Which of the following is the most appropriate diagnostic study to determine the cause of this patient's symptoms? (A) Blood cultures (B) CD4+ T-lymphocyte count (C) Human leukocyte antigen-B27 assay (D) Serum rheumatoid factor assay (E) Stool culture]

**AI_C:** The patient's symptomology as presented points towards reactive arthritis following an infection with Campylobacter jejuni. Reactive arthritis, a type of seronegative spondyloarthropathy, often presents as inflammation in the joints following an infection, especially of the gastrointestinal system. This pathology is associated with the presence of the HLA-B27 antigen, a molecular marker usually found in individuals predisposed to the aforementioned group of diseases including ankylosing spondylitis and psoriatic arthritis. Given that the patient's gastrointestinal symptoms resolved following treatment against Campylobacter jejuni, yet he developed musculoskeletal symptoms afterwards, it makes reactive arthritis a probable diagnosis. Furthermore, his clinical signs such as knee swelling, warmth and pain aggravation with weight bearing, are suggestive of synovitis, a characteristic of reactive arthritis. While septic arthritis, suggested by AI_D, is also a possibility needing a prompt exclusion given its potential severity, it is unlikely in this case due to the absence of persistent fever and absence of noticeable systemic signs of infection. Furthermore, septic arthritis generally does not occur three weeks after the resolution of an infection. Given these conditions, the appropriate diagnostic study would be the HLA-B27 assay (option C). This genetic test would ascertain the presence of the HLA-B27 antigen, a common marker in patients suffering from reactive arthritis. While a negative test does not exclude reactive arthritis, a positive result would support the diagnosis and contribute to the patient's future medical management. In conclusion, understanding the pathogenesis and clinical presentation of reactive arthritis, combined with the knowledge of the patient's recent infection with Campylobacter jejuni, it is advised that the Human Leukocyte Antigen-B27 assay be chosen as the most appropriate diagnostic study. This will aid in confirming the diagnosis of reactive arthritis presenting as a post-infectious complication.

**AI_A:** The primary concern in this patient, as suggested by his ongoing symptoms and preceding infection, appears to be reactive arthritis. Reactive arthritis, a subset of spondyloarthritis, is an immune-mediated inflammatory disorder that typically occurs 1-4 weeks after a gastrointestinal or urogenital infection. The characteristic triad includes arthritis, conjunctivitis/ uveitis, and urethritis, resulting from infections such as those caused by Campylobacter, Shigella, Salmonella, Yersinia, or Chlamydia. Given this patient's recent infection with Campylobacter jejuni and the ensuing arthritis symptoms, the key suspect here is reactive arthritis. While blood cultures, being sensitive to bacterial infections in blood, might help identify a systemic infection leading to possible septic arthritis, it's less likely in this patient considering he responds well to the ciprofloxacin treatment and has no signs of ongoing systemic infection. Alternatively, the HLA-B27 assay would result in information about genetic predisposition to conditions such as reactive arthritis. It should be noted that while up to 80% of patients with reactive arthritis test positive for HLA-B27, its absence doesn't exclude the diagnosis. However, in this patient's case, it would provide valuable insight into his symptomotology. Considering all this patient's circumstances - the recent Campylobacter jejuni infection, the subsequent arthritis without indication of systemic infection, and necessary clinical reasoning, the most appropriate diagnostic study would likely be the human leukocyte antigen-B27 assay (C), as it could reveal a genetic predisposition that, in conjunction with his recent infection, led to this suspected case of reactive arthritis. So, to answer the original question, the Human leukocyte antigen-B27 assay (C) would be the most suitable diagnostic study in this scenario given the patient's presenting symptoms and medical history.

**AI_B:** 1) RESTATING THE QUESTION: This clinical case describes a young man who first had a gastrointestinal illness marked by fever, abdominal pain, and bloody diarrhea, which was caused by Campylobacter jejuni infection. After completion of antibiotic treatment, he developed pain and swelling of his left knee, suggesting arthritic symptoms. The question is which of the available diagnostic studies would be most appropriate to determine the cause of these symptoms. 2) PLANNING: The main assumption is that the patient's joint symptoms might be related to his earlier infection, possibly representing a case of reactive arthritis. An alternate assumption would be that he could be experiencing an unrelated disease, such as rheumatoid arthritis or even septic arthritis. To discern between these possibilities, it seems reasonable to consider testing for Human Leukocyte Antigen B27 (HLA-B27), a genetic marker often associated with reactive arthritis, as well as conducting a blood culture to rule out septic arthritis, and testing for the serum rheumatoid factor to exclude rheumatoid arthritis. Among these, the assumption for reactive arthritis seems to be the best match for the patient's history and symptoms. 3) CLINICAL REASONING: Reactive Arthritis typically manifests in genetically susceptible individuals following bacterial infections, particularly of the gastrointestinal or urogenital tracts, and is associated with HLA-B27 positivity. This patient's age, recent history of Campylobacter jejuni infection, and the current joint symptoms align with a diagnosis of Reactive Arthritis. A blood culture could potentially rule out septic arthritis, but the absence of fever and systemic signs of infection make this unlikely. A serum rheumatoid factor might suggest rheumatoid arthritis, but this condition typically presents as a chronic, symmetrical polyarthritis, which is not the case here. Although the HLA-B27 assay would help establish a predisposition for reactive arthritis, the diagnosis is mainly clinical, and the presence or absence of HLA-B27 does not confirm or refute it. 4) CONCLUDING: Considering the patient's medical history and presenting symptoms, a diagnosis of Reactive Arthritis associated with the Campylobacter jejuni infection seems most likely. While the HLA-B27 assay could provide supporting evidence for this diagnosis, it is not essential for confirmation. 5) RESPONSE: Therefore, based on the most likely clinical reasoning process, the correct answer is (C) Human leukocyte antigen-B27 assay.

**AI_E:** RESTATING THE QUESTION: The situation presents a 23-year-old male patient who was recently treated for a Campylobacter jejuni infection, marked by severe gastrointestinal symptoms. After successfully resolving the infection with ciprofloxacin, the patient began experiencing pain and swelling in his left knee, worsening with weight bearing. The patient's case history and presenting symptoms are suggestive of a post-infection complication, possibly reactive arthritis. The question asks for suggestions on the most appropriate diagnostic test to identify the cause of this patient's current knee symptoms. PLANNING: A few assumptions were made amongst the council members: a majority suggested that the patient is experiencing reactive arthritis as a result of the recent Campylobacter jejuni infection, and the Human leukocyte antigen-B27 assay (HLA-B27 assay) would be the most appropriate diagnostic tool; only one council member suggested a septic arthritis possibility and proposed a blood culture test. To further develop alternate hypotheses, we can revisit these assumptions and question them. Is it possible that the patient has not fully recovered from the infection despite the resolution of its symptoms? Could the ciprofloxacin therapy have caused side effects that are now leading to the patient's knee symptoms? Lastly, could the patient's symptoms be unrelated to previous infection at all, and instead indicative of another systemic or autoimmune disease? CLINICAL REASONING: Reactive arthritis, an inflammation of the joints that occurs after an infection elsewhere in the body, is a common complication of infections by certain types of bacteria including Campylobacter jejuni. However, it is also important to remember that septic arthritis, a potential life-threatening emergency, could present with similar symptoms. The blood culture could help rule out the presence of bacteria in the blood (septic arthritis), however, the lack of systemic symptoms such as fever suggests it is less likely, making this test less clinically significant. If reactive arthritis is suspected, the HLA-B27 assay could validate the assumption but it is also worth noting that it cannot confirm the diagnosis. In addition, ciprofloxacin's commonly reported side effects do not include joint pain or swelling. Considering the patient's age, it is less likely for the symptoms to be representative of systemic or autoimmune diseases like rheumatoid arthritis, which usually presents in older adults and is bilateral in nature, unlike this patient's condition that affects only one knee. CONCLUDING: Following a clinical thought process, it is reasonable to hypothesize that this young patient is likely experiencing reactive arthritis as a post-infection complication from the Campylobacter jejuni infection, making the Human leukocyte antigen-B27 assay a more clinically significant choice for a diagnostic study. While it isn't definitive, it can support the physician's clinical suspicion and aid in the management of the patient's condition. RESPONSE: Given the recent infection and the symptoms presented by this patient, Reactive arthritis is a possible etiological factor to consider, which can be associated with HLA-B27. Therefore, the Human leukocyte antigen-B27 assay (C) would be the most appropriate diagnostic study to determine the cause of this patient's symptoms.

**AI_D:** RESTATING THE QUESTION: The concern here is to determine the most suitable diagnostic study that could explain the young man's symptoms of knee pain and swelling, following an episode of Campylobacter jejuni infection. Reactive arthritis, septic arthritis, rheumatoid arthritis, and other immune conditions such as spondyloarthropathy are all considerations in this clinical scenario. The potential diagnostic options include blood cultures, a CD4+ T-lymphocyte count, a human leukocyte antigen-B27 (HLA-B27) assay, a serum rheumatoid factor assay, and a stool culture. PLANNING: We should consider that reactive arthritis as a consequence of Campylobacter jejuni infection is the most likely differential given the chronology of events. However, to thoroughly assess the situation, It's important to exclude other possible conditions such as septic arthritis which would carry immediate and severe implications if missed, rheumatoid arthritis, or a related immune condition. As such, we must then evaluate which diagnostic tool could provide the most efficient and accurate evaluation. CLINICAL REASONING: It can be tempting to go with the HLA-B27 assay, given that it is known to be associated with spondyloarthropathies such as ankylosing spondylitis and is often seen in patients suffering from reactive arthritis. However, we must note that only a minority of patients with reactive arthritis test positive for HLA-B27 and that a positive result does not confirm the disease nor does a negative rule it out. Contrarily, septic arthritis, which could potentially present with similar symptoms, could have severe, even life-threatening implications if not promptly treated. The gold standard for diagnosing septic arthritis is by joint aspiration, however, blood cultures can be used to identify the causative organism in some cases and thus might be preferred in an initial evaluation given the prognosis of delays in diagnosing septic arthritis. Other options like stool culture and rheumatoid factor assay might seem beneficial, but given the clear report of Campylobacter jejuni from the previous stool culture and the fact that rheumatoid arthritis onset is usually more insidious and bilateral, these options become less viable. CONCLUDING: Although the patient's presentation is suggestive of reactive arthritis due to initial infection by Campylobacter jejuni, ruling out immediate and dangerous differentials like septic arthritis is of clinical priority. A blood culture would aid in this and would more efficiently serve the patient's health outcome whilst still considering a possible reactive arthritis diagnosis that could be dealt with accordingly. RESPONSE: Accordingly, I would opt to initially conduct a blood culture (choice A), as the most appropriate diagnostic study to determine the cause of this patient's symptoms, to promptly rule out septic arthritis, which carries severe implications if diagnosis is delayed, before pursuing other diagnostic assessments for diseases like reactive arthritis.

**Facilitator** **[Round 3]:** Summary: AI_A, AI_B, AI_C, and AI_E all agree that the patient's symptoms suggest reactive arthritis following a Campylobacter jejuni infection. They all recommend the HLA-B27 assay as the most appropriate diagnostic study, as it could reveal a genetic predisposition to conditions such as reactive arthritis. They all acknowledge that while a positive HLA-B27 result does not confirm reactive arthritis, it would provide valuable insight into the patient's condition. AI_D, however, suggests that septic arthritis should be considered due to its severe implications if missed. AI_D recommends a blood culture as the initial diagnostic study to rule out septic arthritis before pursuing other diagnostic assessments for diseases like reactive arthritis. Key Differences: The key difference in the council members' perspectives lies in the prioritization of potential diagnoses and corresponding diagnostic tests. While AI_A, AI_B, AI_C, and AI_E prioritize the likelihood of reactive arthritis and recommend the HLA-B27 assay, AI_D prioritizes the potential severity of septic arthritis and recommends a blood culture. Question for Council Members: Given the patient's symptoms and recent infection, how would you rank the potential diagnoses in terms of likelihood and severity? How does this ranking influence your recommended diagnostic study?. Based on your preceding response, how would you answer the original question: [A 23-year-old man comes to the office because of a 7-day history of fever, crampy abdominal pain, and diarrhea. He says the abdominal pain worsens with oral intake. He has been having 8 to 10 episodes of diarrhea daily. He has not traveled recently and has had no sick contacts. Medical history is unremarkable and he takes no medications. He does not smoke cigarettes, drink alcoholic beverages, or use illicit drugs. He is not sexually active. Vital signs are temperature 38.3°C (101.0°F), pulse 118/min, respirations 18/min, and blood pressure 108/58 mm Hg. Bowel sounds are hyperactive. The abdomen is diffusely tender to palpation; no masses are palpated. Neither the liver edge nor the spleen can be palpated. Digital rectal examination shows grossly bloody stool. A stool culture is obtained, and empiric pharmacotherapy is initiated. Results of the stool culture show growth of Campylobacter jejuni. The patient returns to the office 3 weeks later. He reports total resolution of his fever and gastrointestinal symptoms after completion of prescribed ciprofloxacin therapy, but he has had pain and swelling of his left knee during the past 3 days. The pain worsens with weight bearing. He rates the pain as a 6 on a 10-point scale. Vital signs are temperature 37.1°C (98.8°F), pulse 78/min, respirations 16/min, and blood pressure 112/58 mm Hg. Physical examination discloses erythema and warmth of the left knee with marked synovitis. Direct palpation and flexion of the knee elicit pain. Which of the following is the most appropriate diagnostic study to determine the cause of this patient's symptoms? (A) Blood cultures (B) CD4+ T-lymphocyte count (C) Human leukocyte antigen-B27 assay (D) Serum rheumatoid factor assay (E) Stool culture]

**AI_D:** To answer this question, we need to clarify what the symptoms this patient is presenting with are indicating. Now, the 23-year-old man initially presented with fever, abdominal pain, and diarrhea, which later was identified to be due to Campylobacter jejuni infection. After successful treatment with ciprofloxacin, he has returned with symptoms of pain and swelling of his left knee, which worsens with weight bearing. This progression of symptoms following an infection suggests a condition known as reactive arthritis. For planning, we must take into account several assumptions related with the patient's symptoms. One alternative could be septic arthritis, a severe condition that could occur post-infection. However, the presentation of septic arthritis is typically more acute and may not be accompanied with a complete resolution of primary symptoms, unlike in this case. While it is important to rule out septic arthritis, considering the patient’s specific history and symptom progression, reactive arthritis would be the more clinically prioritized assumption. In terms of clinical reasoning, the onset of joint issues following a bacterial infection provides a solid link to the patient's current condition from his previous infection. Reactive arthritis is an autoimmune condition where the body's immune system "reacts" to a bacterial pathogen by causing inflammation in places far from the initial infection, such as joints. To support this hypothesis, we may want to understand if there is a genetic predisposition for the condition in this patient. For the conclusion, having restated the query, identified the assumptions, and traced the clinical reasoning, we will now take a step-by-step approach to answer our query. Specifically looking for genetic markers associated with autoimmune diseases, particularly arthritis, is important in this context. The most common genetic marker is the HLA-B27 genotype which is often seen in patients with certain types of autoimmune arthritic conditions, including reactive arthritis. In response to the question, the diagnostic test most suited to determine the cause of the patient's current symptoms would be the Human leukocyte antigen-B27 assay (option C). This test would help identify whether or not this patient has a genetic predisposition towards conditions such as reactive arthritis.

**AI_A:** In response to the question, we can start by restating that we have a young, previously healthy man who developed fever, abdominal pain, and diarrhea. A stool culture confirmed Campylobacter jejuni infection, and he was appropriately treated with ciprofloxacin. His gastrointestinal symptoms resolved; however, he subsequently developed pain and swelling in his left knee, which worsened with weight-bearing activities. He displayed signs of synovitis upon physical examination. Now we need to determine the most suitable diagnostic study to evaluate the cause of his joint symptoms. Given the patient's history, one key assumption might be that a post-infectious process is occurring, such as reactive arthritis - a condition that can develop following Campylobacter jejuni infection and presented with joint inflammation. Another possibility could be septic arthritis, which would be a severe disease but less likely given the absence of ongoing systemic symptoms (e.g., no fever). Through clinical reasoning, Reactive arthritis is typically a clinical diagnosis, and no definitive diagnostic criterion exists. As for septic arthritis, it should ideally be identified by analysis of the synovial fluid obtained from the affected joint. Weighing the probabilities, the initial symptoms related to infection with Campylobacter jejuni, and then the appearance of joint symptoms makes a diagnosis of reactive arthritis more likely. The HLA-B27 gene can be found in many patients with reactive arthritis, so testing for this gene could give some additional supporting information – however, it does not confirm the diagnosis, and its absence doesn't rule out the disease. Therefore, considering every aspect, the most appropriate diagnostic measure for this patient would be a joint aspiration and analysis. This method would help to rule out the severe condition - septic arthritis and even gout, which both require urgent management. However, this choice is not among the options given. So, taking into account the given choices, the test for HLA-B27 antigen (option C) would be the best available option. This test would provide some supportive evidence for reactive arthritis, despite its limitations.

**AI_B:** Restating the Question: Essentially, this patient is a 23-year-old male who initially came in with a fever, abdominal pain, and diarrhea. After being diagnosed with a Campylobacter jejuni infection based on the stool culture, he was given ciprofloxacin therapy which resolved his initial symptoms. However, three weeks later, he comes back with pain and swelling in his left knee. The question is asking which is the most suitable diagnostic study to determine the cause of the patient's current set of symptoms. Planning: Reactive arthritis is a post-infectious inflammatory arthritis that typically follows a genitourinary or gastrointestinal infection, such as the one our patient had with Campylobacter jejuni. It is often characterized by the triad: urethritis, conjunctivitis, and arthritis, though not all components may be present. An alternate assumption could be septic arthritis, a severe joint infection that can cause fever, intense joint pain, swelling, and redness. It is important to prioritize the more severe condition, septic arthritis, as it can lead to rapid joint damage if left untreated. Clinical Reasoning: Given the history of the patient's infection with Campylobacter jejuni and the development of monoarthritis weeks later, the most likely diagnosis is reactive arthritis, a condition commonly associated with such infections and presentation. Reactive arthritis often happens a few weeks after a bacterial infection anywhere in the body, commonly Campylobacter in the gut. Nevertheless, the severe joint pain and rapid onset of knee arthritis could also indicate a septic arthritis, a medical emergency that requires prompt identification and treatment to prevent potentially devastating outcomes. Concluding: Even though reactive arthritis is the likely cause of the patient's knee symptoms given the antecedent Campylobacter infection, the differential diagnosis of septic arthritis—a medical emergency—must be ruled out first because of its severity. Therefore, although conducting an HLA-B27 assay may provide valuable information about the patient's susceptibility to conditions like reactive arthritis, it is not the initial diagnostic approach in this clinical setting. Response: Given these considerations, the most appropriate initial diagnostic study to determine the cause of this patient's symptoms would be (A) Blood cultures. Effectively ruling out septic arthritis should be the immediate priority before further investigating other possibilities.

**AI_C:** 1. RESTATING THE QUESTION: The question is asking us to identify the most suitable diagnostic procedure to investigate the symptoms displayed by the 23-year-old patient who initially presented with a history of fever, abdominal pain, and diarrhea, which was later associated with a Campylobacter jejuni infection. Post antibiotic therapy, he is now experiencing pain and swelling in his left knee. The proposed diagnostic options are: blood cultures, CD4+ T-lymphocyte count, human leukocyte antigen-B27 assay, serum rheumatoid factor assay, and stool culture. 2. PLANNING: The question assumes the inflammation in the patient's knee is potentially related to the previous infection. However, two key pathologies that could explain his symptoms are reactive arthritis and septic arthritis. In the case of reactive arthritis, prior exposure to an infection agent - such as Campylobacter - can lead to a subsequent inflammatory response in an 'extraintestinal' site - such as a joint. Alternatively, septic arthritis, which involves direct bacterial invasion of the joint space, may present similarly. The context strongly suggests reactive arthritis, but the clinical consequences of missing septic arthritis could be severely detrimental for the patient. Therefore, we might prioritize ruling out septic arthritis first. 3. CLINICAL REASONING: Reactive arthritis is typically a clinical diagnosis made based on a history of infection followed by inflammatory symptoms developing in the joints, eyes, or urogenital tract. While the human leukocyte antigen-B27 (HLA-B27) assay can show predisposition to certain types of arthritis, it does not confirm the diagnosis of reactive arthritis following Campylobacter infection. On the other hand, septic arthritis is a medical emergency and prompt diagnosis is needed to prevent permanent joint damage. This condition can be confirmed with blood cultures, which can identify the causative bacteria and guide antibiotic selection. 4. CONCLUDING: Given the patient's symptoms of joint inflammation following Campylobacter jejuni infection, a provisional diagnosis of reactive arthritis would be clinically reasonable. However, recognizing the critical importance of excluding potentially severe pathologies promptly, the blood cultures might be most appropriate in this context as they can help to rule out septic arthritis. 5. RESPONSE: Therefore, the most appropriate diagnostic study to determine the cause of this patient's symptoms is (A) blood cultures. Although it seems that reactive arthritis is the most probable explanation, we should first rule out septic arthritis, a medical emergency, before pursuing other diagnostic paths.

**AI_E:** Restating the Question: This scenario involves a 23-year-old male patient who presented with fever, crampy abdominal pain, and frequent diarrhea. The patient's condition improved post-treatment with ciprofloxacin for Campylobacter jejuni infection. However, he returned 3 weeks after initial treatment with new symptoms of pain and swelling in his left knee that worsens with weight bearing. Given the case history, a context-specific query is required on the best diagnostic study to pursue to determine the cause of the patient's new symptoms. Planning: We are required to make assumptions based on the patient's symptom history, initial infection with Campylobacter jejuni, and manifestation of arthritis-like symptoms post-treatment. The leading assumption would be that the patient may have developed reactive arthritis following his intestinal infection. However, considering the patient's presentation, another assumption could be septic arthritis which needs urgent treatment due to its severe prognosis. Additionally, other diseases with similar presentations include HIV infection, ankylosing spondylitis and rheumatoid arthritis. The key here is to decide which diagnostic assumption should be prioritized - reactive arthritis or other conditions. Clinical Reasoning: While the majority of council members leaned towards the diagnosis of reactive arthritis, a small minority have raised the possibility of septic arthritis. Reactive arthritis, previously known as Reiter's syndrome, is typically characterized by the triad of arthritis, conjunctivitis/iritis, and urethritis arriving post-infection (such as Campylobacter) - the arthritis is often pauciarticular and asymmetric with the lower extremities more typically affected than the upper. That's why the HLA-B27 assay is considered, as it's associated with reactive arthritis, but it's noteworthy that a positive test doesn't diagnose the disease, it merely implies an individual's genetic susceptibility. Alternatively, septic arthritis is a severe condition requiring prompt diagnosis and treatment to prevent joint destruction and potentially fatal sepsis. Concluding: After carefully considering the patient's clinical history, examination findings and timing of symptoms, our clinical reasoning is more aligned with the diagnosis of reactive arthritis in response to the C. jejuni infection. Blood cultures are not typically part of the diagnostic pathway for reactive arthritis unless fever or other symptoms of systemic infection are present, so eliminating concurrent septic arthritis is out of our primary concern here. Depending on clinical judgment, an HLA-B27 test may provide useful information on prognosis rather than diagnosis, and the close monitoring of symptoms and clinical progression are of great importance. Response: Based on the presented case history and applying the stepwise reasoning, the most plausible diagnostic study, in this case, might be the 'Human leukocyte antigen-B27 assay' (Option C) to aid further characterization of the reactive arthritis prognosis, however, its interpretation should be carefully considered in the context of specific clinical scenarios and results.

**Facilitator** **[Round 4]:** Summary: AI_A believes that the patient's symptoms suggest reactive arthritis, a condition that can develop following a Campylobacter jejuni infection. They suggest that the most appropriate diagnostic measure would be a joint aspiration and analysis to rule out septic arthritis and gout. However, given the options provided, they recommend testing for the HLA-B27 antigen, which could provide supportive evidence for reactive arthritis. AI_B, AI_C, and AI_E also consider reactive arthritis as the most likely diagnosis but emphasize the importance of ruling out septic arthritis due to its severity. They recommend blood cultures as the most appropriate initial diagnostic study. AI_D, like AI_A, suggests that the patient's symptoms indicate reactive arthritis. They recommend the Human leukocyte antigen-B27 assay as the most suitable diagnostic test to determine if the patient has a genetic predisposition towards conditions such as reactive arthritis. Question to clarify disagreements: Given the differing opinions on the most suitable diagnostic study, could the council members elaborate on why they believe their chosen diagnostic test (HLA-B27 antigen test or blood cultures) is the most appropriate in this case? Specifically, could they discuss the implications of their chosen test in terms of confirming a diagnosis, ruling out other conditions, and guiding treatment?. Based on your preceding response, how would you answer the original question: [A 23-year-old man comes to the office because of a 7-day history of fever, crampy abdominal pain, and diarrhea. He says the abdominal pain worsens with oral intake. He has been having 8 to 10 episodes of diarrhea daily. He has not traveled recently and has had no sick contacts. Medical history is unremarkable and he takes no medications. He does not smoke cigarettes, drink alcoholic beverages, or use illicit drugs. He is not sexually active. Vital signs are temperature 38.3°C (101.0°F), pulse 118/min, respirations 18/min, and blood pressure 108/58 mm Hg. Bowel sounds are hyperactive. The abdomen is diffusely tender to palpation; no masses are palpated. Neither the liver edge nor the spleen can be palpated. Digital rectal examination shows grossly bloody stool. A stool culture is obtained, and empiric pharmacotherapy is initiated. Results of the stool culture show growth of Campylobacter jejuni. The patient returns to the office 3 weeks later. He reports total resolution of his fever and gastrointestinal symptoms after completion of prescribed ciprofloxacin therapy, but he has had pain and swelling of his left knee during the past 3 days. The pain worsens with weight bearing. He rates the pain as a 6 on a 10-point scale. Vital signs are temperature 37.1°C (98.8°F), pulse 78/min, respirations 16/min, and blood pressure 112/58 mm Hg. Physical examination discloses erythema and warmth of the left knee with marked synovitis. Direct palpation and flexion of the knee elicit pain. Which of the following is the most appropriate diagnostic study to determine the cause of this patient's symptoms? (A) Blood cultures (B) CD4+ T-lymphocyte count (C) Human leukocyte antigen-B27 assay (D) Serum rheumatoid factor assay (E) Stool culture]

**AI_E:** Sure, let's put this together. Here the patient presents common symptoms consistent with Arthritis following a gastrointestinal infection due to Campylobacter jejuni. This type of arthritis is typically known as reactive arthritis, which tends to appear following some infections. Reactive arthritis has been strongly associated with the HLA-B27 histocompatibility antigen, a genetic marker. This way, the antigen is an important diagnostic tool when considering reactive arthritis. However, it should be noted that the presence of HLA-B27 is not definitive proof of disease; it just suggests a predisposition. On the other hand, another serious differential diagnosis is septic arthritis, a potentially emergency condition causing joint pain and inflammation due to a bacterial or fungal infection. This could be ruled out by means of a joint aspirate, not listed in the options. So, a blood culture might help to detect the presence of systemic infection to some extent. But, its sensitivity in the detection of septic arthritis is relatively low, making it less reliable. Given this reasoning, from these choices and the history of this patient, it may be more appropriate to conduct an assay for HLA-B27 (Option C). While this will neither definitively diagnose reactive arthritis nor absolutely rule out septic arthritis, it will still provide some additional supporting evidence for a diagnosis of reactive arthritis in the context of the patient's history and clinical presentation. This might help guide the management by confirming the suspicion of reactive arthritis and reducing the possibility of severe differentials like septic arthritis, which should present with more intense clinical symptoms if suspected. Normatively, in live clinical situations, acute septic arthritis (if strongly suspected) needs prompt action with joint fluid sampling and empirical antibiotics even before the reports are available, but our patient has a subacute clinical course with symptoms improving, and that clinical context is not suggestive of septic arthritis. But, this vignette is based on "the following", so from the available options, the assay is the most helpful. So in response to the original question, I believe that the human leukocyte antigen-B27 assay (Option C) would be the most appropriate diagnostic study in this case.

**AI_C:** Restating the question: The 23-year-old patient presented with a 7-day history of fever, abdominal pain, and diarrhea that was later diagnosed as an infection with Campylobacter jejuni. After successful treatment, the patient developed pain and swelling in the left knee, raising new health concerns. An appropriate diagnostic study needs to be identified to determine the cause of his new symptoms. Planning: To address this situation, it's necessary first to consider the nature of reactive arthritis, a condition that often follows bacterial infection and includes symptoms such as inflamed joints, especially at the knee. Given that the patient was diagnosed with a Campylobacter jejuni infection, it is plausible that the patient’s recent joint symptoms may be due to reactive arthritis. Possible assumptions could be that the joint inflammation is due to an independent bacterial infection (possibly septic arthritis), or that it is an auto-immune response following the Campylobacter infection (reactive arthritis). Considering the patient's history, the assumption that the joint inflammation is reactive arthritis, resulting from the Campylobacter infection, seems more probable. Clinical Reasoning: Among the diagnostic options given, the HLA-B27 assay or blood cultures may help to establish reactive arthritis as the diagnosis. If the HLA-B27 antigen is present, it doesn't specifically diagnose reactive arthritis since this antigen is also found in several other rheumatological conditions, and in a proportion of healthy people. However, its presence can provide supportive evidence, especially in a case where reactive arthritis is suspected. Among the other options, blood cultures and stool culture do not seem to be the best fit here. Blood cultures might be necessary if septic arthritis was suspected, but the patient's lack of fever would make this less likely. Likewise, repeating the stool culture may not be particularly helpful since the patient's gastrointestinal symptoms have resolved after treatment. Concluding: In this case, it seems that the HLA-B27 antigen assay might be the most appropriate diagnostic study available among the options, to provide supporting evidence towards a presumptive diagnosis of reactive arthritis. However, this result should be interpreted with caution and in correlation with other clinical findings and investigations as it does not confirm the diagnosis. Response: Given his clinical presentation and history of Campylobacter jejuni infection, the patient's new symptoms are suggestive of reactive arthritis. Therefore, the most appropriate diagnostic study among the options given seems to be the Human leukocyte antigen-B27 assay (C). This test provides supportive evidence, but it does not confirm the diagnosis, and further assessments would likely be necessary.

**AI_B:** RESTATING THE QUESTION: Our patient, a 23-year-old male, who recently recovered from a gastrointestinal infection due to Campylobacter jejuni, presents 3 weeks later with left knee pain, swelling, and evidence of synovitis. The clinical context suggests a possible post-infectious complication. However, the best approach to diagnosing the cause of his pain needs to be determined. PLANNING: There are several possibilities that come to mind, including reactive arthritis (Reiter's syndrome), septic arthritis, or gout. Reactive arthritis commonly manifests after a gastrointestinal or genitourinary infection, the latter of which we can presumably rule out given his sexual history. As part of the diagnostic workup, we should prioritize testing for factors that could influence treatment, prognosis, and contribute to a broader understanding of the patient's presentation. CLINICAL REASONING: Despite the absence of urethritis and conjunctivitis, the symptoms suggest reactive arthritis considering his recent Campylobacter infection. Reactive arthritis usually manifests as an asymmetric oligoarthritis, primarily involving the lower extremities. The best way to confirm this would be through synovial fluid analysis which isn't an option here. Nonetheless, we can potentially rule out a septic arthritis by checking for HLA-B27, common in reactive arthritis and helps identify those at a higher risk for developing spondyloarthopathies. However, It's notable that a negative HLA-B27 does not rule out reactive arthritis. Blood cultures to rule out a septic joint seems reasonable as well, but given the patient's presentation, a bacterial infection seems less likely. CONCLUDING: Given the lack of systemic symptoms, the recent history of Campylobacter infection, and absence of sexual activity, reactive arthritis is the probable diagnosis. RESPONSE: Out of the given choices in this context, the most appropriate diagnostic study to determine the cause of this patient's symptoms would likely be (C) Human leukocyte antigen-B27 assay.

**AI_D:** This medical case revolves around a 23-year-old man who experienced fever, abdominal discomfort, and diarrhea for a week. His symptoms worsened upon eating and his bowel movements numbered 8-10 times daily. He had not been around sick individuals or traveled recently. He led a healthy lifestyle without substance abuse or promiscuity. His vital stats were abnormal and on examination, his abdomen was tender, and he had hyperactive bowel sounds. A rectal exam revealed blood in his stool, prompting stool culture to be taken and precautionary medication begun. The culture eventually returned positive for Campylobacter jejuni. After completing his medication course, his aforementioned symptoms disappeared but he developed swelling and pain in his left knee, which was warm and reddened on examination. Co-existing synovitis accentuated his discomfort. The case presents a complex picture of bowel disturbance followed by joint inflammation. Key in understanding this sequence is the fact that the patient is infected with Campylobacter jejuni, and the subsequent joint pain following successful treatment may suggest reactive arthritis, a condition where the body's immune response to a gastrointestinal or genitourinary infection triggers inflammatory reactions in the joints. The primary priority is to specify through alternate assumption that the joint issue is truly reactive arthritis. When looking at the options given, 'Human leukocyte antigen-B27 assay' is the most viable alternate assumption since it can provide supportive evidence of reactive arthritis. In certain people, presence of the HLA B27 antigen is likely to cause conditions like reactive arthritis, its detection could allude to a predisposition to these. However, it is important to note that an absence of this antigen does not rule out potential auto-immune inflammation. As for 'Blood cultures', while it could be pragmatic in confirming cases of severe infection, given the preceding infection history, they offer little value in isolating Campylobacter jejuni, because bacteremia is very uncommon in patients with Campylobacter enteritis. To summarize, the synthesis of our clinical reasoning is that the patient's symptoms of joint pain post-gastrointestinal complications are suggestive of reactive arthritis, hence the case bears merit for an HLA-B27 assay. This recommendation is founded on the premise that arthritis is a symptomatic manifestation of the systemic immune response activated by an earlier bacterial infection. On the basis of the grounds highlighted, the answer would be: (C) Human leukocyte antigen-B27 assay. This would help in pointing to a diagnosis of Reactive Arthritis, secondary to Campylobacter jejuni infection, given that genetic predisposition is a key motivator of inflammatory reactions seen in Reactive Arthritis.

**AI_A:** The question at hand revolves around determining the best diagnostic study for a 23-year-old male patient experiencing pain and swelling in his left knee. This symptom became noticeable three weeks after an episode of fever and gastrointestinal problems due to an infection by Campylobacter jejuni, which resolved with ciprofloxacin. The current problem reflects marked synovitis in the knee. Restated from a medical perspective, we are seeking a strategic diagnostic approach to understand the etiology of monoarthritis in a previously healthy young man who recently recovered from an enteric infection. Under this context, and before we proceed to steps 2 and 3, let's clarify some key concepts. Monoarthritis refers to inflammation of a single joint, in this case, the left knee. It can arise from various causes, including infection (septic arthritis), autoimmune conditions such as rheumatoid arthritis, crystal-induced arthritis like gout, or it can be a manifestation of systemic diseases such as reactive arthritis. Considering the patient's clinical history and symptomatology, we can assume that this monoarthritis could be a post-infectious phenomenon, specifically reactive arthritis, also known as Reiter's syndrome. This condition is often triggered by a preceding gastrointestinal infection, like Campylobacter, and presents asymmetrically with arthritis, urethritis, and conjunctivitis. However, we should also consider other options, such as septic arthritis or crystal-induced arthritis. Evaluating the choices given, the Human Leukocyte Antigen-B27 (HLA-B27) assay and blood cultures seem to be the most relevant. The HLA-B27 assay is primarily associated with inflammatory conditions like ankylosing spondylitis and reactive arthritis, but it isn't diagnostic on its own. On the other hand, blood cultures could help to identify a bacterial cause if the monoarthritis is septic in origin. Moving to the third step, by systematically connecting these points, if the knee pain is due to reactive arthritis, an HLA-B27 assay could provide supporting evidence but remember, up to 50% of cases of reactive arthritis are positive for HLA-B27. Conversely, if septic arthritis or bacterial infection is a concern, blood cultures, although sometimes negative, could certainly point towards a relevant pathogen causing the knee inflammation. In conclusion, given that reactive arthritis is more likely in the clinical context presented in this case, and it is crucial to confirm or rule out other arthritic conditions, systemically connecting these points, I'd suggest that the Human Leukocyte Antigen-B27 assay would be the most appropriate diagnostic study. This would potentially shed light on a genetic predisposition that could have instigated the development of reactive arthritis post-Campylobacter infection. To respond to the given options: (A) Blood cultures - Less likely, as septic joint is less likely and systemic signs of sepsis are not present. (B) CD4+ T-lymphocyte count - Unnecessary, as HIV, or conditions leading to immunosuppression are not under consideration. (C) Human leukocyte antigen-B27 assay - Most appropriate. Supports diagnosis in favor of reactive arthritis. (D) Serum rheumatoid factor assay - Unnecessary. Rheumatoid arthritis comes with a different clinical demographic and presentation. (E) Stool culture - Unnecessary. The gastrointestinal infection is resolved with therapy.

**Facilitator** The consensus among the AI responses is that the most appropriate diagnostic study for the 23-year-old male patient experiencing pain and swelling in his left knee, following a resolved Campylobacter jejuni infection, would be the Human Leukocyte Antigen-B27 (HLA-B27) assay (Option C). The patient's symptoms suggest a case of monoarthritis, which could be due to various causes such as infection, autoimmune conditions, or systemic diseases. Given the patient's recent gastrointestinal infection, the monoarthritis could be a post-infectious phenomenon, specifically reactive arthritis, also known as Reiter's syndrome. This condition is often triggered by a preceding gastrointestinal infection and presents with arthritis, urethritis, and conjunctivitis. The HLA-B27 assay is associated with inflammatory conditions like reactive arthritis and could provide supporting evidence for this diagnosis. However, it's important to note that the presence of HLA-B27 is not definitive proof of disease; it just suggests a predisposition. While blood cultures could help identify a bacterial cause if the monoarthritis is septic in origin, the lack of systemic symptoms of sepsis in the patient makes this less likely. Other options such as CD4+ T-lymphocyte count, serum rheumatoid factor assay, and stool culture are deemed unnecessary or irrelevant given the patient's clinical history and presentation. In conclusion, the HLA-B27 assay is the most appropriate diagnostic study among the options given, as it could potentially shed light on a genetic predisposition that could have instigated the development of reactive arthritis post-Campylobacter infection. However, this result should be interpreted with caution and in correlation with other clinical findings and investigations as it does not confirm the diagnosis.
